# The effect of a-Lipoic acid (ALA) on oxidative stress, inflammation, and apoptosis in high glucose–induced human corneal epithelial cells

**DOI:** 10.1007/s00417-022-05784-6

**Published:** 2022-09-05

**Authors:** Zhen Li, Yu Han, Yan Ji, Kexin Sun, Yanyi Chen, Ke Hu

**Affiliations:** 1 Department of Ophthalmology, The First Affiliated Hospital of Chongqing Medical University, Chongqing Key Laboratory of Ophthalmology and Chongqing Eye Institute, Chongqing, China; 2 Department of Ophthalmology, The People’s Hospital of Leshan, Leshan, Sichuan Province China; 3 Chongqing Medical University, Chongqing, China

**Keywords:** Diabetic keratopathy, Glycation End Products, Apoptosis, Human corneal epithelial cells, Glucose, Proliferation

## Abstract

**Purpose:**

Oxidative stress and inflammation had been proved to play important role in the progression of diabetic keratopathy (DK). The excessive accumulation of AGEs and their bond to AGE receptor (RAGE) in corneas that cause the formation of oxygen radicals and the release of inflammatory cytokines, induce cell apoptosis. Our current study was aimed to evaluate the effect of ALA on AGEs accumulation as well as to study the molecular mechanism of ALA against AGE-RAGE axis mediated oxidative stress, apoptosis, and inflammation in HG-induced HCECs, so as to provide cytological basis for the treatment of DK.

**Methods:**

HCECs were cultured in a variety concentration of glucose medium (5.5, 10, 25, 30, 40, and 50 mM) for 48 h. The cell proliferation was evaluated by CCK-8 assay. Apoptosis was investigated with the Annexin V- fluorescein isothiocyanate (V-FITC)/PI kit, while, the apoptotic cells were determined by flow cytometer and TUNEL cells apoptosis Kit. According to the results of cell proliferation and cell apoptosis, 25 mM glucose medium was used in the following HG experiment. The effect of ALA on HG-induced HCECs was evaluated. The HCECs were treated with 5.5 mM glucose (normal glucose group, NG group), 5.5 mM glucose + 22.5 mM mannitol (osmotic pressure control group, OP group), 25 mM glucose (high glucose group, HG group) and 25 mM glucose + ALA (HG + ALA group) for 24 and 48 h. The accumulation of intracellular AGEs was detected by ELISA kit. The RAGE, catalase (CAT), superoxide dismutase 2 (SOD2), cleaved cysteine-aspartic acid protease-3 (Cleaved caspase-3), Toll-like receptors 4 (TLR4), Nod-like receptor protein 3 (NLRP3) inflammasome, interleukin 1 beta (IL-1 ß), and interleukin 18 (IL-18) were quantified by RT-PCR, Western blotting, and Immunofluorescence, respectively. Reactive oxygen species (ROS) production was evaluated by fluorescence microscope and fluorescence microplate reader.

**Results:**

When the glucose medium was higher than 25 mM, cell proliferation was significantly inhibited and apoptosis ratio was increased (*P* < 0.001). In HG environment, ALA treatment alleviated the inhibition of HCECs in a dose-dependent manner, 25 μM ALA was the minimum effective dose. ALA could significantly reduce the intracellular accumulation of AGEs (*P* < 0.001), activate protein and genes expression of CAT and SOD2 (*P* < 0.001), and therefore inhibited ROS-induced oxidative stress and cells apoptosis. Besides, ALA could effectively down-regulate the protein and gene level of RAGE, TLR4, NLRP3, IL-1B, IL-18 (*P* < 0.05), and therefore alleviated AGEs-RAGE-TLR4-NLRP3 pathway–induced inflammation in HG-induced HCECs.

**Conclusion:**

Our study indicated that ALA could be a desired treatment for DK due to its potential capacity of reducing accumulation of advanced glycation end products (AGEs) and down-regulating AGE-RAGE axis–mediated oxidative stress, cell apoptosis, and inflammation in high glucose (HG)–induced human corneal epithelial cells (HCECs), which may provide cytological basis for therapeutic targets that are ultimately of clinical benefit.


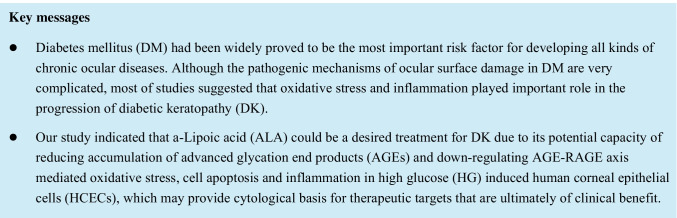


## Introduction

Diabetes mellitus (DM) is a chronic metabolic disease. According to the statistics, there are about 537 million adults (20–79 years) are living with diabetes (1 in 10). This number is predicted to rise to 643 million by 2030 and 783 million by 2045 ^1^. DM had been widely proved to be the most important risk factor for developing all kinds of chronic ocular diseases, such as dry eye disease (DED), delayed epithelial wound healing, corneal edema, recurrent erosions, superficial punctate keratitis, corneal ulcers, and so on ^2^. These problems seem to be affecting up to 70% of diabetic patients ^3^. Although the pathogenic mechanisms of ocular surface damage in DM are very complicated, most of the studies suggested that oxidative stress and inflammation played an important role in the progression of DK ^4–6^.

AGEs are a heterogeneous group of irreversible adducts from glucose-protein condensation reactions, as well as lipids and nucleic acids exposed to reducing sugars. The factors like hyperglycemia or aging have been reported to involve in the generation of AGEs. The bonding of AGEs to their receptors (i.e., RAGE) attributes oxidative stress, which may induce cellular dysfunction and pathophysiological effects. AGEs-mediated ROS generation emerges in the activation of discrete sets of transcription factors and associated genes contributing to various pathological consequences including cardiovascular diseases, cancer, chronic inflammation, neurological disorders and DK ^7–11^. AGE-RAGE axis also appears to have tight relationship in increasing oxidative stress and inflammation during diabetes ^12,13^.

α-Lipoic acid (ALA; 1,2-dithiolane-3-pentanoic acid) also known as thioctic acid, is traditionally recognized as an essential cofactor in mitochondrial respiratory enzymes that catalyze the oxidative decarboxylation reaction ^14^. Due to its powerful antioxidant value, which has been widely used to prevent and treat some metabolic diseases, such as diabetic peripheral neuropathy, reducing plasma cholesterol, protecting liver and heart damage, inhibiting the occurrence of cancer, inhibiting inflammation caused by allergy, arthritis, asthma and anti-aging ^15,16^. However, the effect and molecular mechanisms of ALA on DK were unknown. Therefore, our aim was to evaluate the effect of ALA on AGEs accumulation and study the molecular mechanism of ALA against AGE-RAGE axis mediated oxidative stress, apoptosis and inflammation in HG-induced HCECs, so as to provide cytological basis for the treatment of DK.

## Materials and methods

### Cell culture and treatment

HCECs (Purchased from BNCC, Beijing, China) were cultured in MEM medium (Basal Media, Shanghai, China) supplemented with 10% fetal bovine serum (FBS, Gibco, USA) and Penicillin–Streptomycin Solution (10 kU/ml Penicillin, 10 mg/ml Streptomycin, Procell, Wuhan, China) in a humidified incubator at 37 °C containing 5% CO_2_. According to the groups, D-Glucose (Procell, Wuhan, China) was dilated to different concentration. The whole research procedure was approved by the Ethics Committee of Chongqing Medical University and was performed following the Declaration of Helsinki.

### Cell proliferation assay

The capacity of cell proliferation was evaluated by CCK-8 assay (Shanghai Yi Sheng Biotechnology Co., Ltd., Shanghai, China). This commercially available kit was used following the manufacturer’s instructions. In brief, HCECs (1 × 104 cells per well) were seeded in 96 well plates. The culture medium was replaced after the cells adhered to the wall. The culture medium was replaced with 5.5, 10, 25, 30, 40, and 50 mM glucose for 48 h and then 10 μl CCK-8 solution was added into the microplate. Two hours after incubating, the microplate reader was used to detect the absorbance at 450 nm. According to the results of cell proliferation, 25 mM glucose was used in following HG stimulation. The treating concentration of ALA was determined by the capacity of proliferation. In brief, HCECs (1 × 104 cells per well) were seeded in 96 well plates, the culture medium was replaced after the cells adhered to the wall. The medium was replaced with various concentration of ALA (25, 50, 125, 250, 500 μM, MCE, China) for 24 h at 37 °C in an atmosphere containing 5% CO_2_. According to the cell viability, 25 and 50 μM ALA were used in the following experiment. In order to avoid the influence of osmotic pressure changes caused by high glucose on experimental results, we set 5.5 mM glucose + 22.5 mM mannitol as mannitol hypertonic control. After the HCECs adhered to the wall, the medium was, respectively, replaced with 5.5 mM glucose, 5.5 mM glucose + 22.5 mM mannitol, 25 mM glucose, 25 mM glucose + 25 μM ALA and 25 mM glucose + 50 μM ALA for 24 h at 37 °C in an atmosphere containing 5% CO_2_. 10 μl CCK-8 solution was added into the microplate, 2 h after incubating, the microplate reader was used to detect the absorbance at 450 nm.

### Measurement of intracellular ROS

HCECs were cultured in a 96-well plate with the density of 2 × 10^4^ cells per well. After the cells adhered to the wall, according to the groups, the culture medium was, respectively, replaced with 5.5 mM glucose, 5.5 mM glucose + 22.5 mM mannitol, 25 mM glucose and 25 mM glucose + 25 μM ALA for 24 and 48 h. The production of intracellular ROS was measured with an ROS assay kit (Beyotime Biotechnology, Shanghai, China) using 2′,7′-dichlorodihydrofluorescein diacetate (DCFH-DA) (10 Um) as a fluorescence probe in dark. After incubation with 200 μl per wall diluted DCFH-DA for 30 min at 37 °C, cells were washed three times with PBS. The fluorescence released was detected using a fluorescence microplate reader (Molecular Devices, Sunnyvale, CA, USA) at an excitation and emission wave length of 480 nm and 520 nm. A fluorescence microscope (Leica Microsystems, Wetzlar, Germany) was used to obtain the images. The percentage increase in fluorescence per well was calculated by the formula 【(Ft30-Ft0)/Ft0 × 100】, where Ft30 = fluorescence at time 30 min and Ft0 = fluorescence at time 0 min ^13^.

### Cell apoptosis assay

HCECs were seeded in a six-well plate according to the groups. When the cells adhered to the wall, the culture medium was, respectively, replaced according to the groups. After incubating for 24 and 48 h, cells were harvested and suspended in 1 × Annexin V Bingding Buffer. Then, cells were stained with Annexin V-FITC and propidium iodide (PI) using a cell apoptosis detection kit (Procell Life Scinece & Technology Co., Ltd, China). After incubating for 20 min, cell apoptosis was analyzed by a flow cytometry (BD Biosciences, NJ, USA). The percentage of apoptotic cells was analyzed using FlowJo software (Becton–Dickinson-San Jose, CA, USA). TUNEL cells apoptosis staining was detected by commercial TUNEL cell apoptosis detection Kit (Beyotime, Shanghai, China).

### Protein expression

The protein expression of RAGE, CAT, SOD2, TLR4, NLRP3, Cleaved caspase-3,IL-1β and IL-18 was evaluated by Western blotting (WB). GAPDH was considered as control. In brief, the medium was removed from the six-well plates and cells were washed with iced PBS for three times. The RIPA lysis buffer (Beyotime, Shanghai, China) was used to extract total proteins. Then, a BCA Kit (Beyotime, Shanghai, China) was executed for detecting the concentrations of proteins. A total of 20 μg protein samples in cell lysates were separated in SDS-PAGE gels (FuturePAGE^TM^4%-20%, ACE Biotechnology, Nanjing, China) followed by transferring to polyvinylidene difluoride membranes (Millipore, Bedford, MA, USA). The unspecific bands were blocked with QuickBlock™ Blocking Buffer (Beyotime, Shanghai, China) for 15 min. Then, the membranes were washed with TBST for 15 min. Subsequently, these membranes were, respectively, probed with primary antibodies and stored in 4℃ overnight (RAGE Rabbit pAb, Catalase Rabbit pAb, SOD2 Rabbit mAb, TLR4 Rabbit pAb, NLRP3 Rabbit pAb, Cleaved caspase-3, IL1 beta Rabbit pAb, IL18 Rabbit pAb, GAPDH Rabbit mAb, AB clonal, China). After having been washed with TBST for three times, these membranes were incubated with HRP-conjugated Goat Anti-Rabbit IgG (AB clonal, Wuhan, China). The bands were visualized using an Odyssey Infrared Imaging Scanner (LI-COR Biosciences). Intensity of bands was examined using Image-J software (National Institutes of Health, Bethesda, MA, USA). The protein expression was normalized to GAPDH levels. The cells were lysed with RIPA and the supernatant was collected for detecting the concentration of AGEs by commercially available Enzyme-linked immunosorbent assay (Glucose-derived AGEs ELISA Kit) (Shanghai Xitang Biotechnology Co., Ltd., Shanghai, China).

### Reverse transcription-quantitative polymerase chain reaction (RT-qPCR) analysis

Total RNA was extracted from cells using Trizol reagent (Invitrogen). Then, first-strand cDNA was synthesized from RNA using a Sensiscript RT kit (Takara Biotechnology Co., Ltd., Japan) following the manufacturer’s recommendations. Then, qPCR was conducted with SYBR Green Supermix (Bio-Rad Laboratories, Inc.) on the ABI 7500 system (Applied Biosystems; Thermo Fisher Scientific, Inc.). Relative gene expression was normalized to GAPDH. The relative primers were described in Table 1.

**Table 1:** Nucleotide sequence of specific primers used for polymerase chain reaction amplification (human)

Gene primer	Nucleotide sequence	Gen ID
CAT		847
Forward primer	TGGAGCTGGTAACCCAGTAGG	
Reverse primer	CCTTTGCCTTGGAGTATTTGGTA	
SOD2		6648
Forward primer	GGAAGCCATCAAACGTGACTT	
Reverse primer	CCCGTTCCTTATTGAAACCAAGC	
RAGE		177
Forward primer	ACTACCGAGTCCGTGTCTACC	
Reverse primer	GGAACACCAGCCGTGAGTT	
TLR4		7099
Forward primer	TCCATAAAAGCCGAAAGGTG	
Reverse primer	GATACCAGCACGACTGCTCA	
NLRP3		114,548
Forward primer	CGTGAGTCCCATTAAGATGGAGT	
Reverse primer	CCCGACAGTGGATATAGAACAGA	
IL-18		3606
Forward primer	TCTTCATTGACCAAGGAAATCGG	
Reverse primer	TCCGGGGTGCATTATCTCTAC	
IL-1B		3553
Forward primer	ATGATGGCTTATTACAGTGGCAA	
Reverse primer	GTCGGAGATTCGTAGCTGGA	
GAPDH		2597
Forward primer	GGAGCGAHATCCCTCCAAAAT	
Reverse primer	GGCTGTTGTCATACTTCTCATGG	

### Immunofluorescence

HCECs were seeded in 24-well plates according to the groups. When the cells adhered to the wall, the culture medium was, respectively, replaced according to the groups. After incubating for 48 h, the HCECs were fixed by 4% PFA for 10 min, rinsed with PBS to remove PFA, then incubated for 12 min in 1% Triton X-100 (Aladdin, Shanghai, China). After being washed, cells were incubated in 3% BSA for blocking. The permeabilized cells were incubated with primary antibody overnight at 4 °C. Then, after being served with PBS again, cells were incubated with goat anti-rabbit IgG (H + L), fluorescein Isothiocyanate conjugate (TransGen, Beijing, China) for 1 h at room temperature and DAPI for 7 min. Cover the cell slides on the micro slides with Mounting Medium, antifading, and around the cell slides with neutral balsam to prevent drying. Specimens were examined with a Leica SP8 Laser Scanning confocal microscope (Leica, Wetzlar, Germany).

### Statistical analysis

Representative data from three independent experiments are presented as means ± standard deviation (SD). A significant difference between two groups using Mann–Whitney *U* test/Student’s test and more than two groups by One-way ANOVA followed by Bonferroni’s multiple comparison test using Graph pad prism 8 (Graphpad Holdings, LLC, San Diego, CA, USA). Statistical significance was defined as *p* < 0.05.

## Results

### Effect of HG stimulation on cell proliferation and apoptosis

**Fig. 1:**
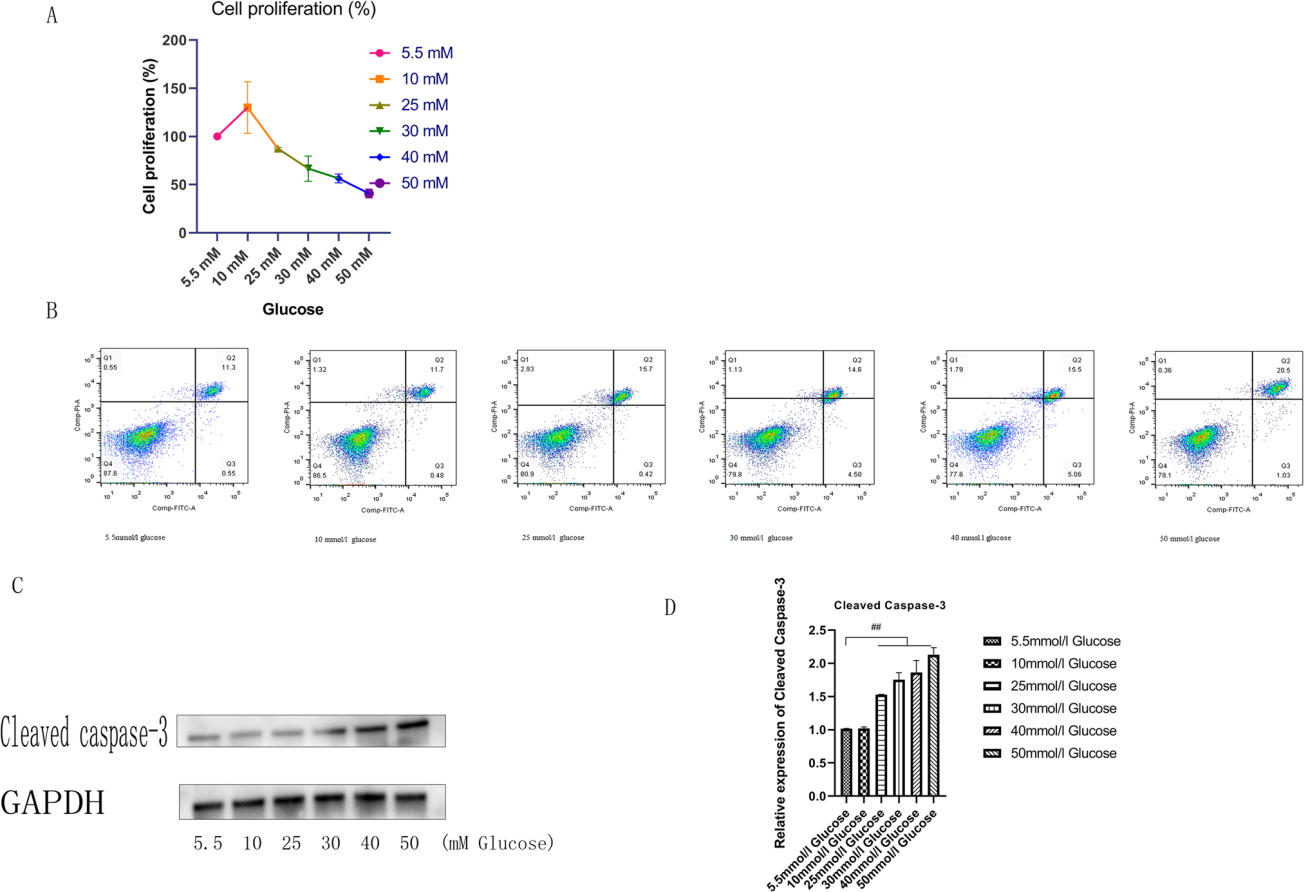
**Effect of high glucose on cell proliferation and apoptosis.** (**A**) The cell proliferation of HCECs was evaluated by CCK-8 assay. Data are the mean ± SD of three independent experiments for all groups. The cell proliferation ratio (%) of HCECs in 5.5 mM glucose medium was 130.05 ± 26.77, in 25 mM glucose medium was 87.374 ± 1.157. (**B**) Cells apoptosis was evaluated by Flow cytometry. From 25 mM glucose to 50 mM glucose, the total apoptosis rate of HCECs was gradually increased from 16.1 to 21.53%. Q3 = early apoptosis, Q2 = late apoptosis, total apoptosis = Q2 + Q3. (**C** and **D**) The expression of apoptosis-related proteins cleaved caspase-3 was evaluated by Western blotting. GAPDH was considered as control. Data shown were from three independent experiments. Compared to 5.5 mM glucose medium group, from 25 to 50 mM glucose medium, the cleaved caspase-3 protein expression of HCECs was significantly up-regulate. “##” *P* < 0.001

From the result of Fig. 1A, we found that when the concentration of glucose medium reached to 25 mM, the cell proliferation rate (%) of HCECs had been decreased to 87.374 ± 1.157. However, compared with 5.5 mM glucose medium, HCECs treated with 10 mM glucose showed a higher cell proliferation rate (%), which was increased to 130.05 ± 26.77. The results of the above indicated that slightly high glucose environment was favorable for cell proliferation, but when the glucose medium concentration was higher than 25 mM, cell proliferation would be remarkably inhibited (*P* = 0.00046). From the results of flow cytometry (Fig. 1B), we got insight that the number of apoptosis of HCECs was increased after the stimulation with 25 mM glucose. From 25 to 50 mM glucose, the total apoptosis rate of HCECs was gradually increased from 16.1 to 21.53%. Concurrently, the expression of apoptosis-related proteins cleaved caspase-3 were evaluated by Western blotting. As presented in Fig. 1C and D, compared to 5.5 mM glucose medium group, from 25 to 50 mM glucose medium, the cleaved caspase-3 protein expression of HCECs was significantly up-regulated (*P* < 0.0001). The data suggested that glucose medium higher than 25 mM would inhibit HCECs proliferation and increase cell apoptosis. Therefore, 25 mM glucose was used in the following HG experiments.

### Effect of ALA on cell proliferation in HG-induced HCECs

**Fig. 2:**
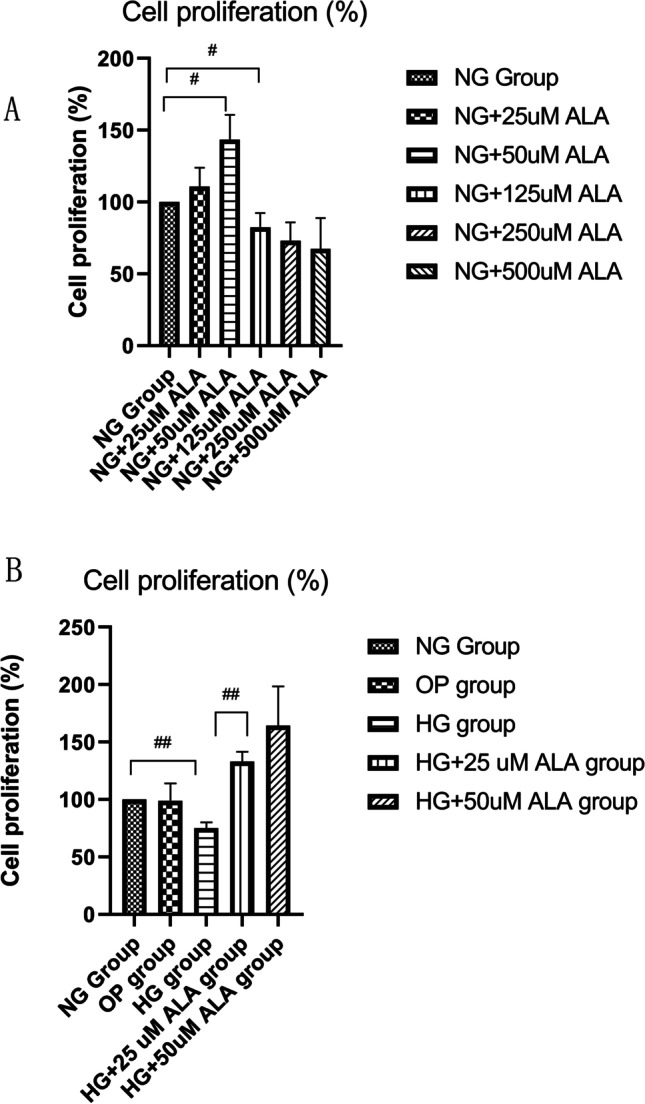
**Effect of ALA on cell proliferation.** (**A**) Compared to normal glucose medium without ALA, administration of 50 uM ALA presented a notably effect on the cell proliferation. However, the administration of 125 uM ALA presented a remarkable inhibition on cell proliferation. Data shown were from three independent experiments. “#” *P* < 0.05. (**B**) Compared to HG group, the administration of 25 uM ALA presented significant effect on promoting cell proliferation. Data shown were from three independent experiments. “##” *P* < 0.001

After treatment with different doses of ALA (25, 50, 125, 250, and 500 μM) in normal glucose medium for 24 h, the effect of ALA on cell proliferation was detected by CCK-8 assay. From the result of Fig. 2A, we found that compared to normal glucose medium without ALA, the administration of 50 μM ALA presented a notable effect on the HCECs proliferation (*P* = 0.002). However, the administration of 125 μM ALA presented a remarkable inhibition on cell proliferation (*P* = 0.013). In the following experiment, both 25 and 50 μM ALA were used to investigate the cell proliferation in HG environment. As exhibited in Fig. 2B, compared to HG group, the administration of 25 μM ALA presented significant effect on promoting cell proliferation (*P* < 0.001).

### Effect of ALA on accumulation of AGEs and expression of RAGE in HG-induced HCECs

**Fig. 3:**
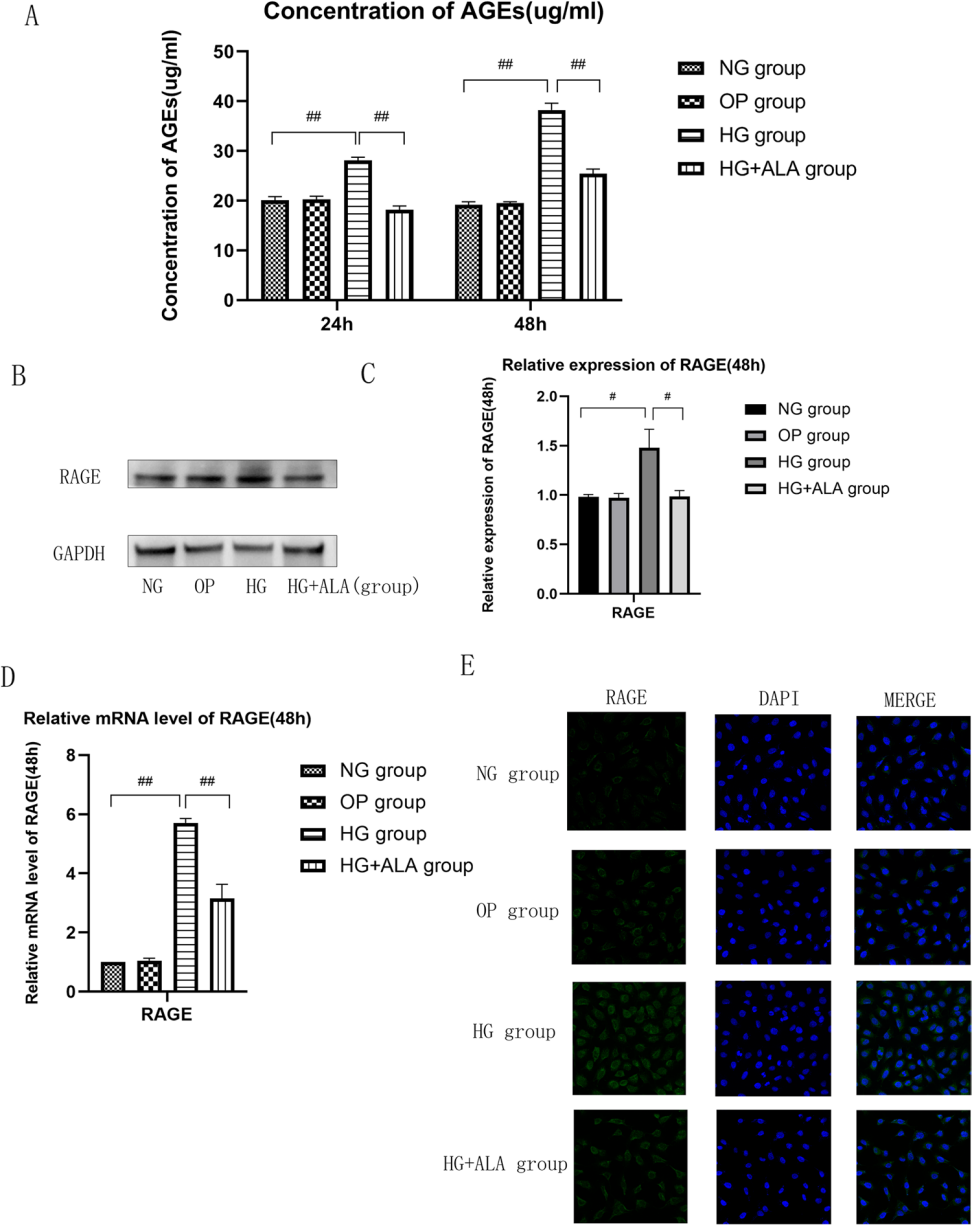
**Effect of ALA on accumulation of AGEs and expression of RAGE in HECEs.** (**A**) The intracellular concentration of AGEs was detected by commercially available ELISA Kit. Data were from three independent experiments for all groups. Compared to NG group, the production of AGEs in HG group was significantly increased after the stimulation of 25 mM glucose for 24 and 48 h (*P* < 0.001), but which could be significantly inhibited by the treatment of 25 uM ALA. “##” *P* < 0.001. (**B** and **C**) The relative expression of RAGE protein was evaluated by Western blotting. GAPDH was considered as control. Data were from three independent experiments for all groups. Compared to NG group, the protein expression of RAGE was significantly increased in HG group, however, the administration of ALA significantly down-regulated protein expression of RAGE in high glucose medium. “#” *P* < 0.05. (**D**) The relative expression of RAGE mRNA was evaluated by RT-qPCR. GAPDH was considered as control. Data were from three independent experiments for all groups. Compared to NG group, AGER mRNA was significantly up-regulated in HG group. However, compared to HG group, the administration of ALA significantly down-regulated the mRNA level of AGER in HG + ALA group. “##” *P* < 0.001. (**E**) Captured immunofluorescent staining images of RAGE. DAPI and their merged images were also demonstrated (scale bar = 400 µm)

As presented in Fig. 3A, compared to NG group, the production of AGEs in HG group was significantly increased after the stimulation of 25 mM glucose for 24 (*P* < 0.001) and 48 h (*P* < 0.001), but which could be significantly inhibited by the treatment of 25 uM ALA (*P* < 0.001). The concentration of AGEs in HG group and HG + ALA group was positively correlated with the treatment time. We were surprised to observe that the administration of 25 μM ALA significantly alleviated the accumulation of AGEs and down-regulated protein expression of RAGE (*p* = 0.012) in HG induced HCECs (Fig. 3B and C). The mRNA level of RAGE was further confirmed by RT-PCR (Fig. 3D). In HG-induced HCECs, 25 μM ALA treatment significantly down-regulated the mRNA expression of RAGE (1.86-fold, *p* = 0.00085). The same results also were observed in immunofluorescence images (Fig. 3E). Collectively, our results suggested that ALA treatment could alleviate the accumulation of AGEs, down-regulate the expression of RAGE in HG-induced HCECs.

### Effect of ALA on oxidative stress in HG-induced HCECs

**Fig. 4:**
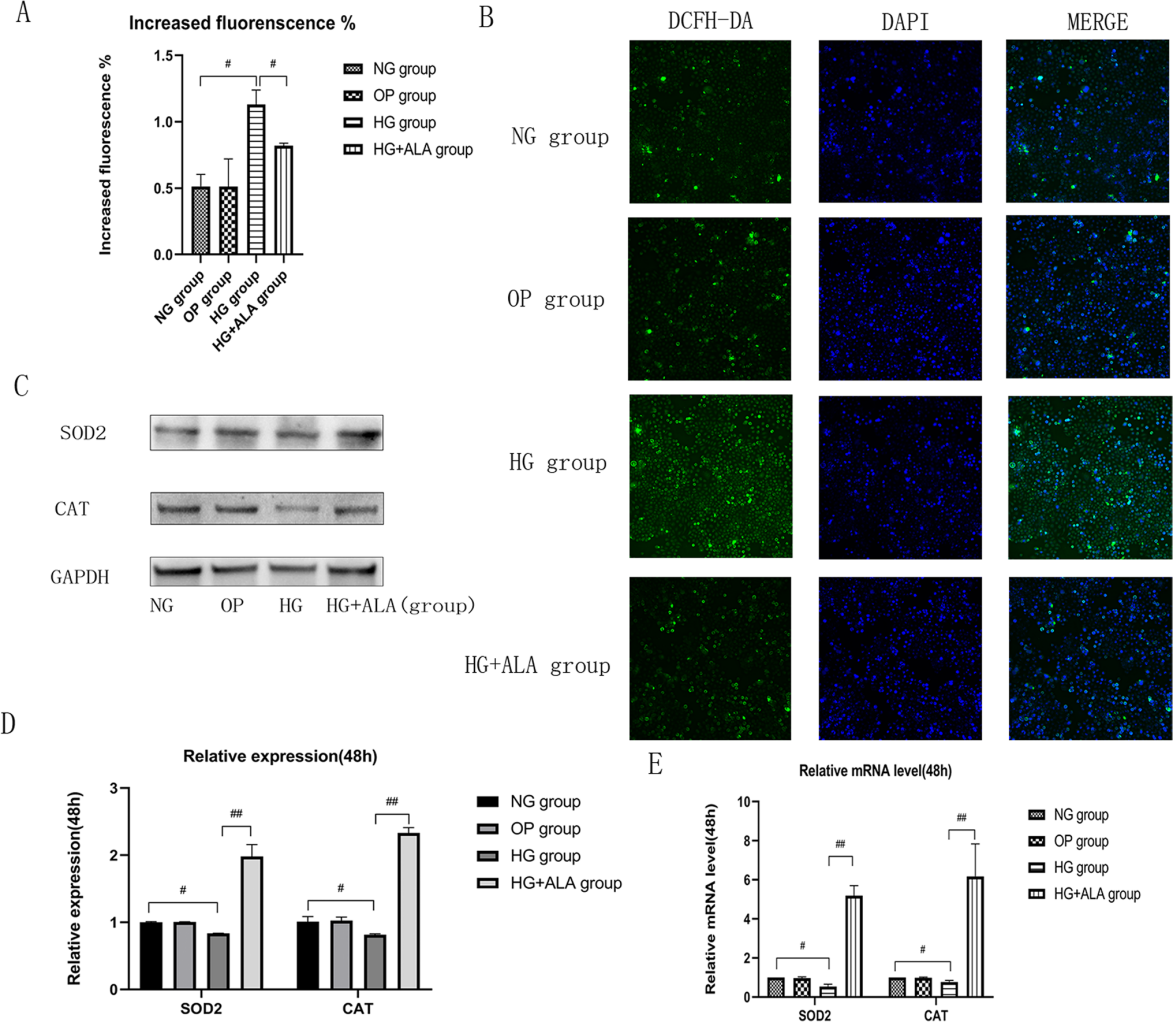
**Effect of ALA on oxidative stress in high glucose induced HCECs.** (**A** and **B**) Intracellular ROS was detected with an ROS assay kit ((DCFH-DA). ROS positive cells were stained with green fluorescence (scale bar = 100 µm). Data were from three independent experiments for all groups. Compared to NG group, the increased fluorescence was significantly increased in HG group. However, the administration of ALA significantly inhibited the increased fluorescence. “#” *P* < 0.05. (**C** and **D**) The relative protein expression of CAT and SOD2 was evaluated by Western blotting. GAPDH was considered as control. Data were from three independent experiments for all groups. Compare to NG group, the relative protein expression of CAT and SOD2 was significantly inhibited in HG group. However, the administration of ALA could significantly activate the expression of CAT and SOD2 in high glucose induced HCECs. “#” *P* < 0.05, “##” *P* < 0.001. (**E**) The relative expression of AGER mRNA was evaluated by RT-qPCR. GAPDH was considered as control. Data were from three independent experiments for all groups. Compared to NG group, CAT and SOD2 mRNA was significantly down-regulated in HG group. However, compared to HG group, the administration of ALA significantly up-regulated the mRNA level of CAT and SOD2 in HG + ALA group. “#” *P* < 0.05, “##” *P* < 0.001

To confirm the protective effect of ALA on oxidative stress induced by HG stimulation, the production of intracellular ROS, the oxidative stress–related proteins and genes were detected. From the results of Fig. 4A and B, we found that the increased fluorescence in HG group (*P* = 0.017) was obviously higher than that in NG group, but the increased fluorescence could be obviously alleviated after the treatment of ALA (*P* = 0.008). Meanwhile, we found that compared to NG group, the relative expression of oxidative stress–related CAT(*P* = 0.014) and SOD2 (*P* = 0.011) protein was obviously decreased in HG group. We also observed that the CAT (*P* = 0.002) and SOD2 (*P* = 0.008) mRNA level was significantly down-regulated. But the protein and gene expression of CAT (*P* < 0.001) and SOD2 (*P* < 0.001) could be significantly activated after the treatment of ALA (Fig. 4C to E). These results demonstrated that ALA could inhibit the formation of ROS, activate the protein and gene expression of CAT and SOD2, and therefore alleviated oxidative stress induced by HG stimulation.

### Effect of ALA on cell apoptosis in HG-induced HCECs

**Fig. 5:**
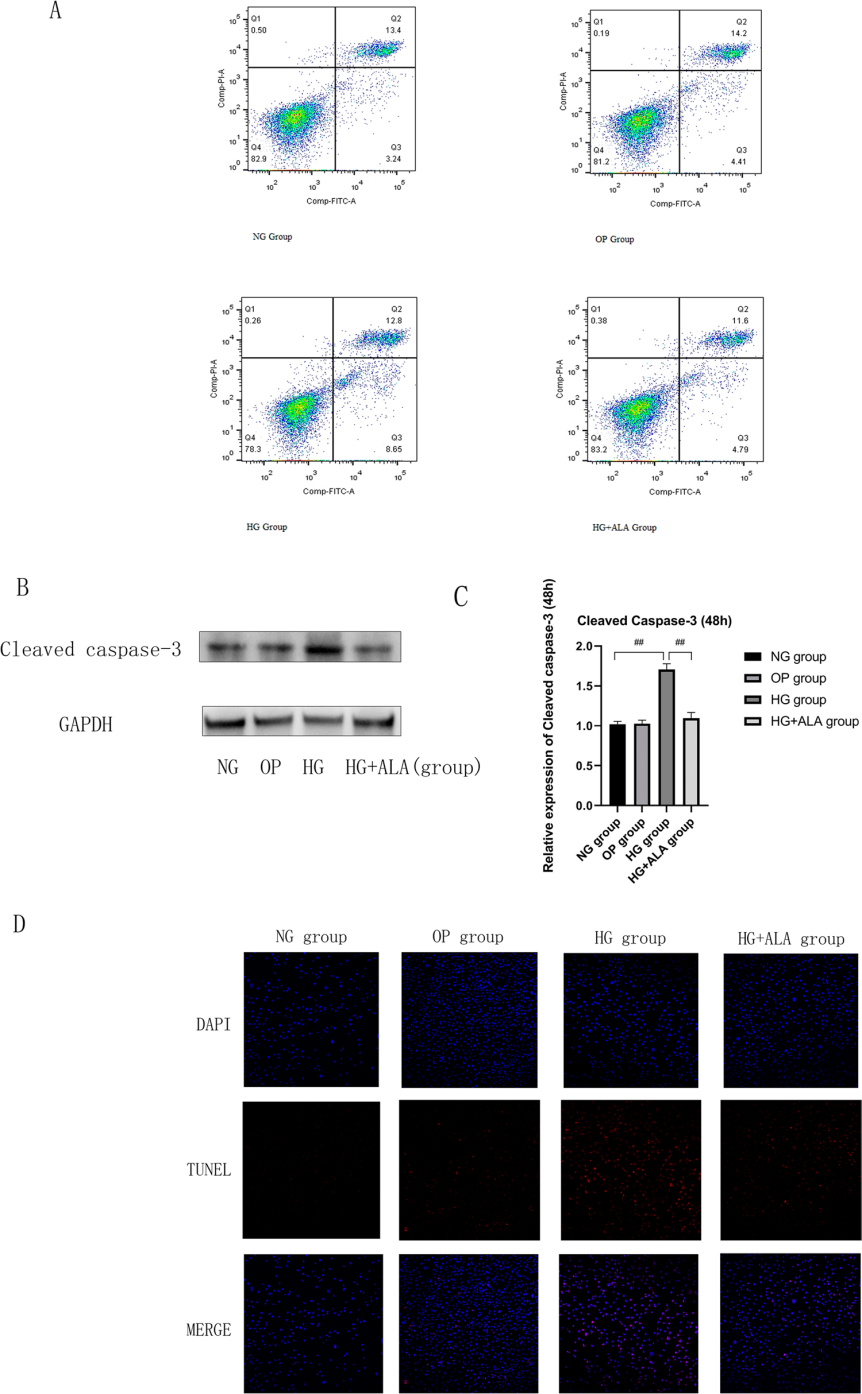
**Effect of ALA on cell apoptosis in high glucose induced HCECs.** (**A**) In HG group, the early apoptotic cells accounted to 8.65% and the total apoptotic cells accounted to 21.45%. However, after the administration of ALA, the cell apoptosis ratio was decreased, especially the early cell apoptosis was decreased from 8.65 to 4.79%. Q3 = early apoptosis, Q2 = late apoptosis, total apoptosis = Q2 + Q3. (**B** and **C**) The protein expression of apoptosis-related cleaved caspase-3 was evaluated by Western blotting. GAPDH was considered as control. Data were from three independent experiments for all groups. Compared to NG group, the relative protein expression of cleave caspse-3 was significantly increased in HG group. However, compared to HG group, the relative expression of cleave caspse-3 protein could be remarkably down-regulated by the treatment of ALA. “##” *P* < 0.001. (**D**) TUNEL staining was used to detect cell apoptosis. DAPI (blue), TUNEL positive staining (red), scale bar = 100 µm

As presented in Fig. 5A, we found that compared to NG group, the early and total apoptotic cells in HG group were increased after the stimulation of HG. In HG group, the early apoptotic cells ratio accounted to 8.65% and the total apoptotic cells ratio accounted to 21.45%. But, after the treatment of ALA, the cell apoptosis ratio was decreased, especially the early cell apoptosis was decreased from 8.65 to 4.79%. The result of cleaved caspase-3 protein expression also proved that ALA treatment could have significantly inhibited HG-induced HCECs apoptosis (*P* < 0.001). (Fig. 5B and C). TUNEL apoptotic cells staining also confirmed the similar results to flow cytometry (Fig. 5D).

### Effect of ALA on inflammation in HG-induced HCECs

**Fig. 6:**
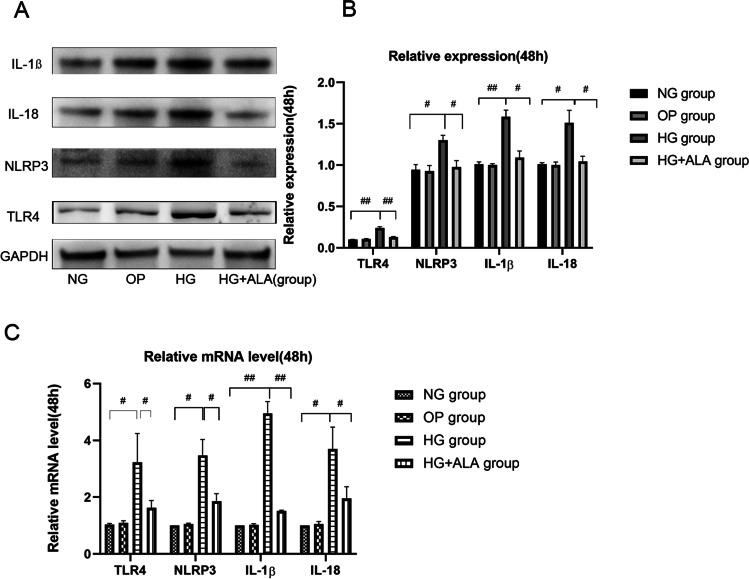
**Effect of ALA on inflammation in high glucose induced HCECs.** (**A** and **B**) The protein expression of TLR4, NLRP3, IL-1β and IL-18 was evaluated by Western blotting. GAPDH was considered as control. Data were from three independent experiments for all groups. Compared to NG group, the relative protein expression of TLR4, NLRP3, IL-1β and IL-18 was significantly increased in HG group. However, compared to HG group, the relative protein expression of TLR4, NLRP3, IL-1β and IL-18 could be remarkably down-regulated by the treatment of ALA. “#” *P* < 0.05, “##” *P* < 0.001. (**C**) The relative expression of TLR4, NLRP3, IL-1β and IL-18 was evaluated by RT-qPCR. GAPDH was considered as control. Data were from three independent experiments for all groups. Compared to NG group, TLR4, NLRP3, IL-1β and IL-18 mRNA were significantly up-regulated in HG group. However, compared to HG group, the administration of ALA significantly down-regulated the mRNA level of TLR4, NLRP3, IL-1β and IL-18 in HG + ALA group. “#” *P* < 0.05, “##” *P* < 0.001

**Fig. 7:**
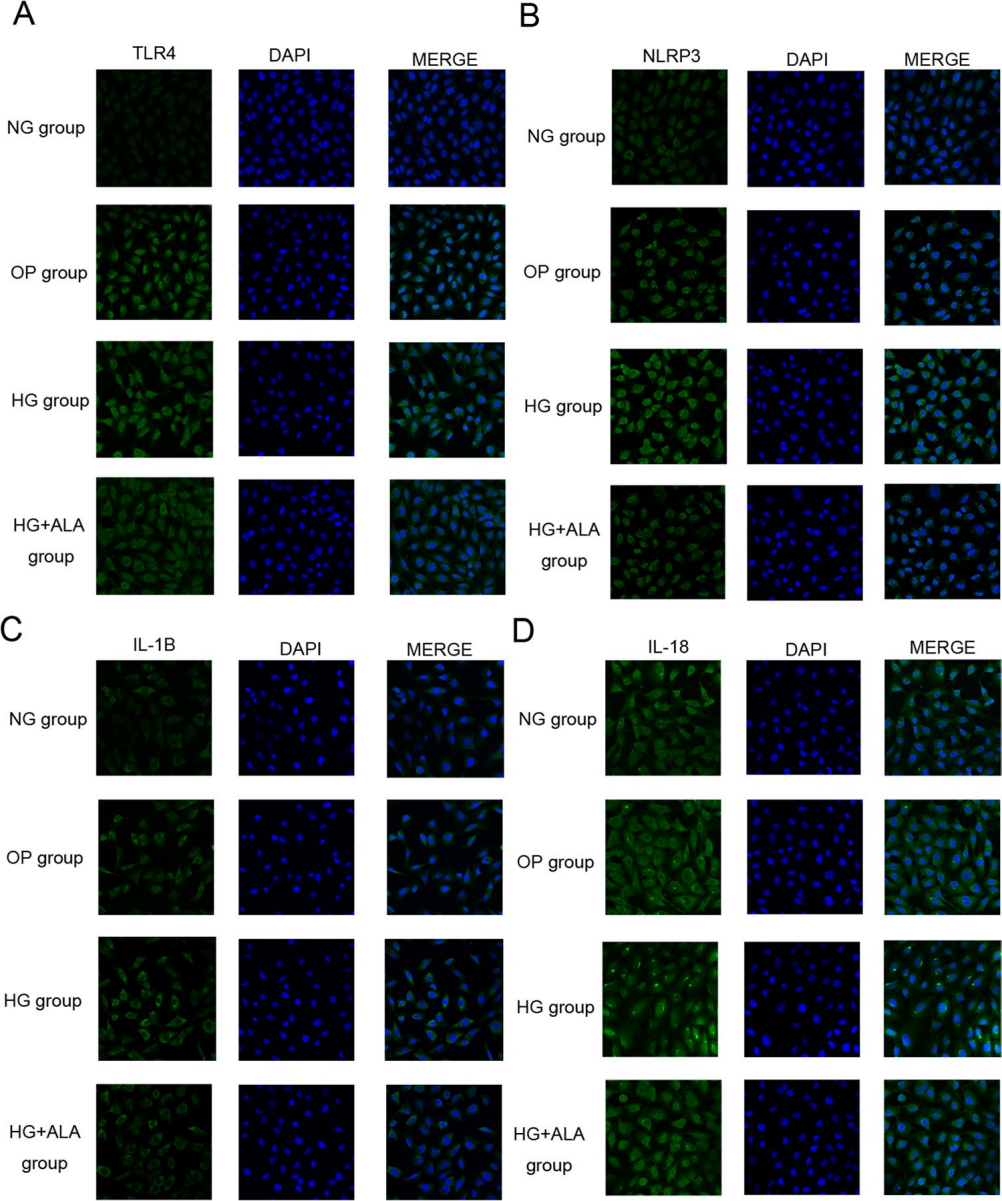
**Immunofluorescent staining images.** (**A**) Captured immunofluorescent staining images of TLR4. DAPI and their merged images were also demonstrated (scale bar = 400 µm). (**B**) Captured immunofluorescent staining images of NLRP3. DAPI and their merged images were also demonstrated (scale bar = 400 µm). (**C**) Captured immunofluorescent staining images of IL-1β. DAPI and their merged images were also demonstrated (scale bar = 400 µm). (**D**) Captured immunofluorescent staining images of IL-18. DAPI and their merged images were also demonstrated (scale bar = 400 µm)

In order to evaluate the effect of ALA on inflammation in HG stimulation, we detected the protein and gene level of TLR4, NLRP3, IL-1β and IL-18 in HG induce HCECs. As presented in Fig. 6A and B, we found that compared to NG group, the relative protein expression of TLR4 (*P* < 0.001), NLRP3 (*P* = 0.002), IL-1ß (*P* = 0.0003) and IL-18 (*P* = 0.005) was significantly increased in the HG group. However, the administration of ALA presented significant effect on down-regulating the expression of TLR4 (*P* < 0.001), NLRP3 (*P* = 0.005), IL-1ß (*P* = 0.002) and IL-18 (*P* = 0.008). Meanwhile, the results of RT-PCR (Fig. 6C) also proved that ALA could effectively down-regulate HG-induced mRNA level of TLR4 (*P* = 0.0181), NLRP3 (*P* = 0.01), IL-1ß (*P* = 0.001) and IL-18 (*P* = 0.025). The same results also could be observed in immunofluorescence images (Fig. 7). Hence, we speculated that ALA could play an anti-inflammatory role through down-regulation of TLR4-NLRP3 signaling pathway.

## Discussion

Diabetes is a public health problem that concern all countries in the world. DK, the most frequent clinical condition affecting the human cornea, is a potential sight-threatening condition caused mostly by epithelial disturbances that are of clinical and research attention due to its severity. Diabetic keratopathy exhibits several clinical manifestations, including persistent corneal epithelial erosion, superficial punctate keratopathy, delayed epithelial regeneration, and decreased corneal sensitivity that may lead to compromised visual acuity or permanent vision loss ^17^.

A large number of studies have proved that sustained hyperglycemia causes non-enzymatic glycosylation of various proteins in vivo and AGEs are important in the pathogenesis of chronic complications of diabetes ^18^. Besides the diabetic complications, AGEs also play important role in many widespread age-related pathology such as Alzheimer’s disease, decreased skin elasticity ^19–21^, male erectile dysfunction ^22,23^ and atherosclerosis promotion, progression, and prevention ^24^. AGEs can accumulate in and out of cells and is therefore associated with a variety of eye diseases, such as diabetic retinopathy ^25^, cataract ^26^, glaucoma ^27^, age-related macular degeneration ^28^, dry eye ^29^, diabetic keratopathy ^30^, and so on. The pathogenic mechanism involved may be mainly through non-receptor–mediated and receptor-mediated. In the process of AGEs formation, proteins are modified to promote cross-linking between proteins, resulting in abnormal structure and function of corresponding organ or cells. The glycosylation of collagen and elastin cloud weakens the compliance of tissues and organs, while the glycosylation of laminin and fibronectin could affect cell adhesion, migration and differentiation. Glycosylation of enzymes, receptors and DNA molecules in cells could affect normal physiological functions and metabolic activities, resulting in cell degeneration and death. AGEs also can bind to RAGE and therefore induce local cell signaling towards apoptotic and proliferative pathways, as well as increases oxidative stress and inflammation. In our study, we evaluated the intracellular concentration of AGEs and the expression level of RAGE, we found that the concentration of AGEs and the relative expression level of RAGE were significantly higher in HG group, the concentration of AGEs in cells was positively correlated with the duration of HG stimulation. We still found that the HCECs viability would be significantly inhibited, while the ratio of apoptosis would be greatly increased in HG stimulation. Besides the above results, we also observed that HG stimulation would increase the accumulation of ROS and induce up-regulating of TLR4, NLRPE, IL-1B, IL-18 in HCECs. We speculated that HG stimulation could increase the accumulation of AGEs and accelerate AGEs binding to RAGE, which would activate ROS production, and therefore induce apoptosis, up-regulate TLR4 signaling pathway and activate inflammatory cytokine release in HCECs. These speculations were consistent with some former reports ^31,32^.

It is likely that reducing the accumulation of AGEs and inhibiting AGEs binding to RAGE may be a potential treatment strategy for diabetic keratopathy. ALA is a kind of multifunctional antioxidant with chemical structure of 6, 8-dilipoic acid, which can be worked as a coenzyme to participate in the acyl transfer in the metabolism of substances in our body and eliminate free radical–induced aging and disease. The potential antioxidant effect of ALA can be ascribed to the direct reactive oxygen species (ROS) scavenging capacity, metal ion chelating ability and ability to restore the cellular antioxidants such as reduced glutathione (GSH), coenzyme Q, vitamins C and E levels. In laboratory experiments, the effect of ALA on protein glycation and AGEs formation had been investigated both in vitro and in vivo. Dietary supplementation of ALA in rats fed chronically with glucose significantly decreased mitochondrial superoxide in the heart and AGEs formation in the aorta ^33^. Supplementation of ALA in fructose-fed-rats significantly attenuated AGEs-mediated skin-collagen crosslinking and other physicochemical abnormalities ^34^. ALA could protect against fructose-mediated myoglobin glycation in vitro by inhibiting the early and intermediate glycation reactions involved in the formation of AGEs and therefore ALA supplementation is beneficial in the prevention of AGEs-mediated diabetic and cardiovascular complications ^35^. At the cellular level, ALA is reduced to dihydrolipoic acid (DHLA), which has a number of cellular actions including free radical scavenging and modulating oxidative stress and inflammatory pathways ^36^. Moreover, ALA could markedly suppress AGEs-induced activation of NF-kB in cultured vascular endothelial cells and in retinal endothelial cells ^37^. Exogenous administration of ALA diminished AGEs-induced endothelial expression of vascular cell adhesion molecule-1 (VCAM-1) and monocyte binding to endothelium ^38^. Furthermore, ALA prevented the up-regulation of AGEs-induced inducible nitric oxide synthase (iNOS) expression and nitric oxide (NO) production in murine microglial cells ^39^. In the field of eye diseases, ALA had been successfully employed in a variety of in vivo models, such as diabetic retinal vascular lesions ^40^, cataract ^41^, glaucoma^42^, and diabetic corneal diseases^43^, all of which include complex and intimate association between increased oxidative stress and increased inflammation ^44^. However, none of the previous studies had focused on the effects of ALA on AGEs accumulation and the molecular mechanisms on AGE-RAGE signaling pathway in HG-induced HCECs.

In our study, we observed that 25 μM ALA showed a significant effect on inhibiting AGEs accumulation and down-regulating RAGE expression in HG-induced HCECs. The possible mechanism of action of ALA in inhibiting the formation of AGEs might include (a) blocking the amino groups of protein, thus preventing its glycation with free sugar, (b) blocking the carbonyl groups of reducing sugars, (c) blocking the Amadori products and dicarbonyl intermediates which may reduce glycation, as well as AGEs formation, preventing autoxidation of fructose and glycoxidation. Mechanistic studies on the effects of ALA on the redox status of insulin-responsive cells revealed that ALA stimulated glucose uptake by affecting components of the insulin-signaling pathway and prevented excesses glucose converting to AGEs ^45^. Besides, we were surprised to find that the ROS production was significantly inhibited after the treatment of ALA, meanwhile, the oxidative stress–related CAT and SOD2 expression was significantly activated. In the study of AM et al. ^46^, they also proved the similar results. In HG group, the early apoptotic cells accounted to 8.65% and the total apoptotic cells accounted to 21.45%. However, after the treatment of ALA, the cell apoptosis ratio was obviously decreased, especially the early cell apoptosis was decreased from 8.65 to 4.79%. We speculated that the anti-apoptotic effect of ALA was mainly through inhibiting AGEs accumulation and RAGE binding, thus inhibiting AGE-RAGE-ROS pathway–induced cell apoptosis.

In our study, we also studied the anti-inflammatory effects of ALA on HG-induced HCECs. We were delighted to find that ALA had predominant effect on down-regulating the expression of TLR4, NLRP3, IL-1β and IL-18. Some previous studies had reported that AGEs could bind to RAGE or up-regulate the expression of RAGE, and therefore activate TLR4, NLRP3, FOXC2, pPKCβ 1, JNK, p38 MAPK and NF-κB pathways ^12,32,47–51^. All of the results were related to extracellular and intracellular oxidative stress and inflammation. Hu et al. ^52^ had demonstrated that TLR4-NLRP3 pathway plays a critical role in the inflammation and apoptosis of retinal ganglion cells induced by high glucose. Garibotto et al. ^53^ also proved that TLR4, nucleotide-binding oligomerization domain-containing protein 2 (NOD2), and NLRP3 inflammasome are involved in the production and persistence of inflammation in diabetic nephropathy. In the study of DK, Wan et al. ^54^ studied the effect of the NLRP3 inflammasome on diabetic corneal wound healing and never regeneration. They proved that NLRP3 inflammasome–mediated inflammation and pyroptosis contributed to DK pathogenesis. They also revealed that the accumulated AGEs promoted hyperactivation of the NLRP3 inflammasome through ROS production, genetically and pharmacologically blocking the AGEs/ROS/NLRP3 inflammasome axis significantly expedited diabetic corneal epithelial wound closure and nerve regeneration. Therefore, we speculated that the anti-inflammatory effects of ALA on HG-induced HCECs were probably through blocking the AGEs-RAGE-TLR4-NLRP3 axis. However, the specific signaling pathway of ALA against mediated oxidative stress, inflammation and apoptosis is still worthy of further study.

## Conclusion

It is likely that reducing the accumulation of AGEs and inhibiting AGEs binding to RAGE may be a potential treatment strategy for diabetic keratopathy. Our study indicated that ALA could be a desired treatment for diabetic keratopathy due to its potential capacity of alleviating AGEs accumulation, inhibiting AGEs-RAGE-ROS–mediated oxidative stress and cells apoptosis, as well as down-regulating AGEs-RAGE-TLR4-NLRP3 axis–induced inflammation. Understanding the mechanisms of ALA on HG-induced HCECs may provide cytological basis for therapeutic targets that are ultimately of clinical benefit.

## Data Availability

Available upon request from the first author: Dr. Zhen Li.

## Abbreviations

AGEs: Advanced glycation end products
AGER: Advanced glycation end product receptor
ALA: A-Lipoic acid
HCECs: Human corneal epithelial cells
NG: Normal glucose
HG: High glucose
OP: Osmotic pressure
CAT: Catalase
DK: Diabetic keratopathy
SOD2: Superoxide dismutase 2
Cleaved caspase-3: Cleaved cysteine-aspartic acid protease-3
NLRP3: Nod-like receptor protein 3
IL-1 ß: Interleukin 1 beta
IL-18: Interleukin 18
ROS: Reactive oxygen species
DM: Diabetes mellitus
DED: Dry eye disease
MAPK: Mitogen-activated protein kinase
BSA: Bovine serum albumin
